# Effect of the micro-environment on α-synuclein conversion and implication in seeded conversion assays

**DOI:** 10.1186/s40035-019-0181-9

**Published:** 2020-01-17

**Authors:** Niccolo Candelise, Matthias Schmitz, Katrin Thüne, Maria Cramm, Alberto Rabano, Saima Zafar, Erik Stoops, Hugo Vanderstichele, Anna Villar-Pique, Franc Llorens, Inga Zerr

**Affiliations:** 1 Department of Neurology, University Medicine Goettingen and the German Center for Neurodegenerative Diseases (DZNE), Robert-Koch -Straße 40, 37075 Göttingen, Germany; 2 Biomedical Engineering and Sciences Department, School of Mechanical and Manufacturing Engineering (SMME), National University of Sciences and Technology (NUST), Islamabad, Pakistan; 3 Department of Experimental, Diagnostic and Speciality Medicine, University of Bologna, Bologna, Italy; 4 Departamento de Neuropatología y Banco de Tejidos (BT-CIEN), Fundación CIEN, Instituto de Salud Carlos III Centro Alzheimer Fundación Reina Sofíac, Valderrebollo n° 5, 28031 Madrid, Spain; 5 ADx NeuroSciences, Technologiepark 4, Ghent, Belgium; 6 CIBERNED (Network center for biomedical research of neurodegenerative diseases), Institute Carlos III, Madrid, Spain; 7 Bellvitge Biomedical Research Institute (IDIBELL), Hospitalet de Llobregat, Barcelona, Spain

**Keywords:** RT-QuIC, α-Synucleinopathies, Protein misfolding, Protein strains

## Abstract

**Background:**

α-Synuclein is a small soluble protein, whose physiological function in the healthy brain is poorly understood. Intracellular inclusions of α-synuclein, referred to as Lewy bodies (LBs), are pathological hallmarks of α-synucleinopathies, such as Parkinson’s disease (PD) or dementia with Lewy bodies (DLB).

**Main body:**

Understanding of the molecular basis as well as the factors or conditions promoting α-synuclein misfolding and aggregation is an important step towards the comprehension of pathological mechanism of α-synucleinopathies and for the development of efficient therapeutic strategies. Based on the conversion and aggregation mechanism of α-synuclein, novel diagnostic tests, such as protein misfolding seeded conversion assays, e.g. the real-time quaking-induced conversion (RT-QuIC), had been developed. In diagnostics, α-synuclein RT-QuIC exhibits a specificity between 82 and 100% while the sensitivity varies between 70 and 100% among different laboratories. In addition, the α-synuclein RT-QuIC can be used to study the α-synuclein-seeding-characteristics of different α-synucleinopathies and to differentiate between DLB and PD.

**Conclusion:**

The variable diagnostic accuracy of current α-synuclein RT-QuIC occurs due to different protocols, cohorts and material etc.. An impact of micro-environmental factors on the α-synuclein aggregation and conversion process and the occurrence and detection of differential misfolded α-synuclein types or strains might underpin the clinical heterogeneity of α-synucleinopathies**.**

## Background

α-Synucleinopathies, such as Parkinson’s disease (PD), dementia with Lewy bodies (DLB) or multiple system atrophy (MSA), are a class of neurodegenerative diseases characterized by the presence of misfolded, fibrillary α-synuclein. In PD and DLB, the deposits of α-synuclein (Lewy bodies, (LBs)) occur intracellular within neurons, while MSA patients show oligodendroglial cytoplasmic inclusions (GCI), called Papp-Lantos bodies, rather than neuronal aggregates.

Even though the cellular distribution of aggregated α-synuclein is different, there is a significant pathological overlap. In both diseases, neurodegeneration is associated with the LBs and GCI burden, as well as the increase of soluble α-synuclein in substantia nigra and striatum [1]. In addition, there is an apparent overlap in degeneration of the dopaminergic nigrostriatal system in PD and MSA. The folding process of a protein (e.g. α-synuclein) occurs in dependence on its energetic landscape, defined by the thermodynamic constrictions that force it to assume the conformation of the lowest free energy. Whereas protein folding is regulated mostly by chaperones [2, 3], a class of proteins called intrinsically disordered proteins (IDPs, such as α-synuclein), which owns a low complexity region that may adopt an alternative, β-sheets enriched conformation [4–7], show poor binding features to chaperones [8–11]. IDPs may form fibrils and plaques [12], characterized by a β-sheet conformation with β-strands perpendicular to the length of the fibril, favoured by an exclusion of water from the inner core of the fibril and hydrophobic interactions. The summation of all forces enables the misfolded protein to cross its metastable intermediate stages and lean toward the minimal energy level of the misfolded amyloid [13]. Whereas equilibrium among the native and the intermediate stages (which may be populated by oligomers) is possible, the formation of amyloids represents an irreversible process. Previous studies indicate that the misfolding and subsequent conversion of one or more proteins into a pathogenic variant may contribute to the onset of neurodegenerative diseases, retracing the protein-only hypothesis originally formulated for the prion protein [14–17]. According to this view, the propagation of misfolded proteins requires the formation of a homotypic complex between the two conformers of a protein [17], which leads to the conversion of the native form into the pathogenic form. The novel β-sheet-enriched molecule may act as a seed, contacting other native molecules (typically referred as substrate) and imposing the conversion into β-sheets, establishing a positive feedback that eventually causes the aggregation into plaques and fibrils.

In this review we focus on α-synuclein, a protein whose intracellular aggregates are hallmarks in α-synucleinopathies. At first, we discuss the potential prion-like conversion behaviour of α-synuclein. Afterwards, we summarize the micro-environmental factors that may affect the α-synuclein aggregation process and their impact on the diagnostic performance of major techniques based on the seed-mediated conversion of misfolded species of α-synuclein. At last, we review the state of the art about the existence and the impact of different synuclein strains that may contribute to different clinical features in α-synucleinopathies.

## Main text

### Conversion and transmission of misfolded α-synuclein

Being a natively unfolded protein [18], α-synuclein aggregation is ruled by thermodynamics [19]. The first event in the assembly of α-synuclein is supposed to be dimerization [20], leading in turn to the generation of aggregation-competent oligomers, encompassing a wide variety of structures [21], and possessing their own kinetics of fibrillization and toxicity [22].

The conversion behaviour of α-synuclein, described as “prion-like”, has been reported by evidences of graft-to-host spreading of α-synuclein aggregates [23–25]. Aggregated α-synuclein can be transmitted from neuron to neuron [26] either after neuronal death or by exocytosis-mediated pathways [26], while the uptake may occur be endocytic pathways [27], substantiating the neuron-to-neuron transmission and the predictable spreading observed in some forms of α-synucleinopathies. Recently, α-synuclein was shown to interact with high density lipoproteins [28] and the presence of α-synuclein, both in its native and misfolded conformation, was found in extracellular vesicles as well [29, 30], suggesting a potential novel route for the spreading of the pathology. Moreover, α-synuclein transmission along axonal connections was demonstrated ex vivo and in vivo in various animal models.

*Luk* and co-workers demonstrated that fibrillary α-synuclein is able to trigger the conversion of monomeric α-synuclein into aggregates closely reminiscent of LBs and neurites in a cell model overexpressing α-synuclein [31]. Fibrillary α-synuclein was further found to be transported anterogradely through the axon and taken up by the next neuron in mouse embryonic cultures [32], while in vivo studies conducted with rats showed the trans-neuronal spreading of fibrils from the olfactory bulb toward other non-olfactory regions [33]. The transport of fibrillary α-synuclein forms were shown to follow an anterograde pathway through the axon and passaged to the next neuron in mouse embryonic cultures. Moreover, in vivo studies conducted in rats demonstrated that α-synuclein fibrils spread from the olfactory bulb toward other non-olfactory regions [33]. Others showed that α-synuclein aggregates were inoculated in the medulla oblongata of rats, and the caudo-rostral spreading was followed by showing fibers and neuronal abnormalities in the rostral mesencephalon and in prosencephalic regions after 18 weeks [34]. Lastly, PD pathology was successfully passaged to non-human primates by administration of human PD-derived LBs.

The spreading of α-synuclein through different routes may highlight the differential affinity of different cell types toward α-synuclein binding and internalization, as the cellular milieu differs among populations of cells derived from different developmental lineages. Thus, the cellular environment may play an instructive role for the spreading of misfolded α-synuclein, affecting first highly vulnerable cell lineages.

In the next section, we will address the environmental factors that may affect the aggregation of α-synuclein, its seeding ability and thus the modes of spreading through various routes.

### Micro-environmental factors affecting the α-synuclein aggregation

A plethora of environmental factors have been found to affect protein misfolding and aggregation [35]. Physical factors, such as molecular crowding and temperature, may affect protein aggregation by enhancing the entropy of the system. Similarly, chemical factors such as pH variations or the presence of other proteins or biomolecules, such as nucleic acids, phospholipids and proteoglycans, may affect the aggregation process. Moreover, post-translational modifications (PTMs) may also interfere with the aggregation process by affecting the surface charge or the topology of the protein. The effect of these conditions on α-synuclein aggregation is summarized in Table 1.

**Table 1:**
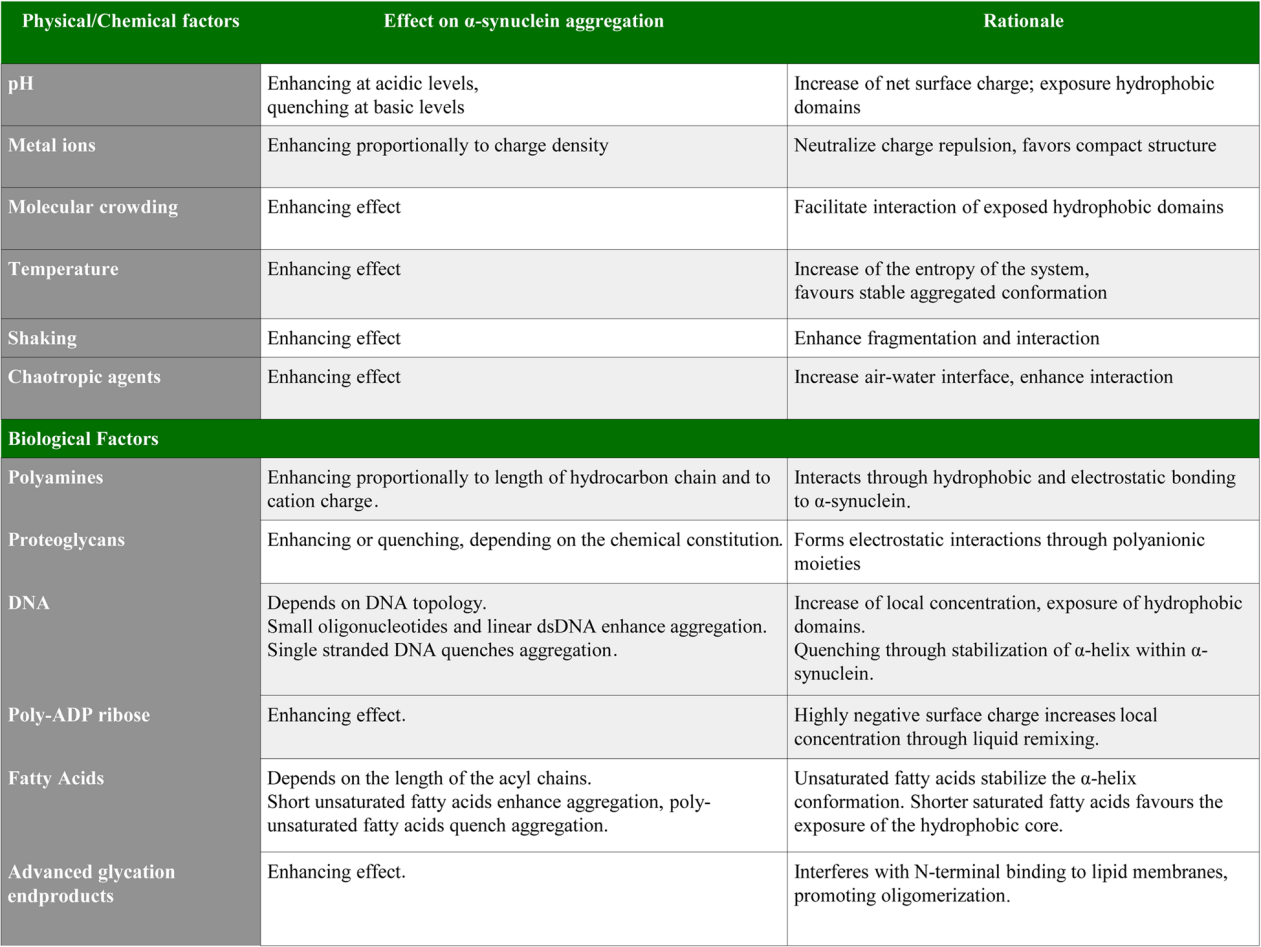
Factors affecting α-synuclein aggregation. The table summarizes the effects of various chemical, physical and biological agents on α-synuclein aggregation. Effect is either defined as enhancing when promotes the aggregation or quenching when it slows down the aggregation. The rationale for the effect is listed in the table as well.

Additional, often overlooked factors in PD patients, which influence the misfolding and aggregation features of α-synuclein, are missense point mutations in the α-synuclein gene (SNCA) (e.g. A53T, A53E) as well as the potential increase of α-synuclein expression levels, induced by duplications or triplications of SNCA. The genetic A53T missense mutation causes conformational changes in α-synuclein with structural alterations of the β-sheet structures, thermodynamically more stable in aqueous solutions, exhibiting a significant influence on the aggregation process [36]. Experimentally, the integration of a genetic PD associated point mutation in recombinant α-synuclein substrate would accelerate the seeding response of oligomeric α-synuclein in seeded aggregation assays. However, it may also promote the self-aggregation of the substrate and the generation of false-positives, as we discuss in a dedicated section below.

#### PH and metal ions

Low pH can be viewed as a factor promoting protein aggregation by increasing the net surface charge and hence enhance side chain repulsion, allowing the exposure of the aggregation-prone hydrophobic core [37]. Indeed, the growth of α-synuclein fibrils was shown to be impaired at neutral pH while it was magnified at mildly acidic pH values (5.8) [38], indicating a critical role of the chemical conditions of the micro-environment surrounding the nascent fibril. Similarly, changes in the environmental pH have been found to affect the amyloidogenic properties of β2-microglobulin, a protein expressed in nucleated plasmatic cells whose aggregates are found in dialysis-related amyloidosis at acidic pH levels [39]. Metal ions have been shown to behave similarly to pH variations by neutralizing charge repulsion and thus imposing a more compact and aggregation-prone structure to α-synuclein [40]. The efficiency in inducing the folding increases with the charge density, reaching a maximum at approximately 2.5 Å^− 3^. Therefore, small polyvalent cations are more effective in triggering the structural changes when compared to large monovalent cations. Polyvalent cations may also create bridges between carboxylate residues, further increasing the transition to a partially folded structure.

#### Polyamines

Polyamines are a group of physiological polycations involved in several biological functions, spanning from DNA replication to protein synthesis [41]. They present multivalent cations separated by aliphatic hydrocarbon chains, through which they can interact with natively unfolded proteins both via electrostatic and hydrophobic interactions [42]. High order polyamines, such as putrescine, spermine and spermidine, have been found to be involved in cellular toxicity during the development of PD [43]. Polyamines have been shown to enhance α-synuclein aggregation in a manner proportional to the cation charge and the length of the aliphatic chains separating the amino groups [44]. Therefore, the most potent among them is spermine, which owns four cationic groups separated by three hydrocarbon chains. An in-depth study of the conformational change of α-synuclein induced by spermine has been carried out using force spectroscopy coupled with atomic force microscopy [45]. Here, the C-terminal alanine of α-synuclein has been substituted with a cysteine, thus allowing the covalent binding to the tip of the atomic force microscope and to the mica surface. With this experimental design, researchers were able to show that α-synuclein assembly is potently enhanced by polyamines at the very first steps of the process, indicating that even the first steps of the aggregation (i.e., dimerization) may show heterogeneity.

#### Proteoglycans

Proteoglycans are glycoproteins containing sulphated glycosaminoglycan chains (GAG). They are expressed in the outer leaflet of the cellular membrane, where they are involved in the internalization of amyloids [46, 47]. Due to the presence of both a negatively charged moiety (sulphated groups) and a positively charged amino acids, their binding to amyloids has been reported to be both detrimental and protective [48, 49] and, consequently, different types of proteoglycans may display opposite effects towards protein aggregation.

Agrin, a neuronal heparin sulphate proteoglycan, has been found to localize with all Alzheimer’s lesions and has been proposed to contribute to the formation of LBs and neurites. Binding studies and circular dichroism analyses have shown that agrin increases the insolubility of α-synuclein in vitro and enhances the formation of the fibrils [50]. Since agrin contains two potential sites for the attachment of GAG moieties, a subset of glycosylated agrins may exist in the brain, each owing different functions and distinct affinities for α-synuclein.

#### Nucleic acids

Nucleic acids have been reported in association to amyloid plaques and tangles in protein misfolding diseases. Since they own high binding affinity for amyloids, they have been supposed to act as a scaffold to promote protein aggregation [51]. Due to the net positive surface charge of amyloids, the favoured electrostatic interactions of the peptide chain and phosphate groups of nucleic acids promote the aggregation. This interaction may further increase the local concentration of amyloid proteins, supporting the aggregation through interaction of the exposed hydrophobic domains with neighbour proteins [52, 53].

Nucleic acids have been indeed shown to enhance prion protein, superoxide dismutase-1, amyloid-β and tau aggregation [54]. α-Synuclein has been shown to possess differential binding affinity towards different conformations of nucleic acids. *Hegde* and co-workers [55] studied the binding of α-synuclein with different topologies of DNA by circular dichroism spectroscopy, intrinsic fluorescence and binding to fluorescent reporter for amyloids such as thioflavin-T (Th-T) and ANS. Single-stranded circular DNA was shown to induce the formation of an α-helix within α-synuclein, inhibiting the aggregation, whereas small oligonucleotides and linear double-stranded DNA caused a partial folding that leans towards the aggregated state. Moreover, GC-rich DNA was shown to induce aggregation in a more efficient way compared to AT-rich DNA.

#### Poly-ADP ribose (PAR)

Poly-ADP ribose (PAR) is a non-coding polynucleotide produced by the PAR polymerase upon DNA damage [56], mediating a form of necrosis termed parthanathos (from the ancient Greek embodiment of death, *Thanathos*), which represents a major cell death pathway in neurodegenerative diseases [57]. Outside of triggering cell death through the mitochondrial release of the apoptosis initiating factor [58], PAR was shown to accumulate next to the DNA breakage foci upon external or internal stressful events, where it initiates liquid remixing to achieve intracellular, membrane-less compartmentalization [57]. Such newly formed organelles act as a seed for the assembly of IDPs, which are in turn excluded from damaged areas to allow DNA reparation and promote genome stability. Depletion of PARP by CRISPR-Cas9 (Clustered Regularly Interspaced Short Palindromic Repeats and CRISPR-associated protein 9) in neuronal cultures was shown to prevent cell death mediated by α-synuclein pre-formed fibrils (PFFs) and to inhibit their propagation to neighbouring cells [59]. Indeed, α-synuclein PFFs were shown to trigger PARP activation via production of nitric oxide and subsequent DNA damage. In turn, PAR overexpression caused enhanced α-synuclein aggregation through liquid remixing, producing α-synuclein strains that were more compact and hence more toxic. These results were replicated in vivo, showing that whereas intrastriatal injection of α-synuclein PFFs in wild type recipient mice caused increased PAR levels and loss of dopaminergic neurons within 6 months, injection in PARP-1 knockout recipient mice failed to trigger neuronal loss.

#### Fatty acids

Lipid bilayers have been indicated as a factor that may reduce the activation energy barrier required for proteins to accommodate into the amyloid state [60], by interacting with the positively charged surface of amyloidogenic proteins and thus increase the local concentration and unmask the hydrophobic core of the proteins. Many neurodegenerative related proteins, including α-synuclein [61], have been shown to insert to lipid bilayers [60] and have been suggested to act as anchor for fibrils assembly. Indeed, the interaction with biological membranes may increase the local concentration of the unfolded protein, thus increasing the probability of exposing the hydrophobic core that drives protein aggregation. *Perrin* and co-workers [62] analyzed the exposure of α-synuclein to different fatty acids. Roughly two thirds of α-synuclein forms a vesicle binding domain, which was proposed to serve a role in synaptic physiology [63]. Upon binding to vesicles, α-synuclein is reshaped into an α-helix containing conformation, which was shown to be resistant to various disruption methods, such as SDS, acidification and boiling. However, not every lipid can trigger α-synuclein aggregation, as the length of the acyl chain is critical for the aggregation. Only short and saturated hydrocarbon chains [64] were shown to be able to induce α-synuclein aggregation, whereas poly-unsaturated fatty acids [65] form stable multimers with α-synuclein. Therefore, it appears that α-synuclein aggregation may either be enhanced or quenched according to the kind of fatty acid it binds to. Lastly, the physical properties of the membrane also affect the aggregation, as it was shown that α-synuclein binding to biological membranes is much stronger when it binds to the fluid state compared to the gel phase [66].

#### Post-translational modifications (PTMs)

The interaction of α-synuclein with the micro-environment is influenced by the presence of PTMs, which may change the degree and the type of interaction, resulting either in enhancing or quenching the kinetic of aggregation. Indeed, the presence of phosphorylated and ubiquitinated α-synuclein is a hallmark of LBs and neurites [67]. Although the role of PTMs in synucleinopathies is more complex and has been extensively reviewed elsewhere [68, 69], we briefly summarized the most relevant PTMs in the process of α-synuclein aggregation.

Clinical and functional studies [70–72] converge on the observation that the dysregulation of glucose metabolism may be an important risk factor for the development of α-synuclein related pathologies. Catabolic processing of glucose generates advanced glycation end-products (AGEs), reducing sugars that may covalently bind to proteins, thus affecting their structure and function. By displaying a comprehensive approach involving yeast models, human stem cells, flies and mice models, and human tissues, *Vicente Miranda* and co-workers [73] demonstrated by a wide variety of biochemical, biophysical imaging and electrophysiological techniques that glycation of α-synuclein in its amino-terminal moiety by AGEs exploits α-synuclein toxicity and aggregation, impairs motor ability and electrophysiological properties. These effects appear to be mediated by the disruption of the N-terminal binding to lipid membranes, altering the trafficking and hindering the ubiquitination, thus impairing the degradation through the proteasomal pathway.

These evidences suggest that hyperglycemia may contribute to the onset of synucleinopathies, substantiating the role of diabetes as a risk factor for Parkinson’s disease. Moreover, the association of AGEs with α-synuclein aggregation corroborates the association between mutations in genes coding for glyoxalase and deglycase enzymes, such as DJ-1 and recessive forms of Parkinson’s disease.

Among the various phospho-sites involved in α-synuclein aggregation, phosphorylation at residue Ser129 appears to be the most relevant in pathology and, therefore, the most studied [74]. However, data diverges in identifying the role of this PTM in pathology, as it was shown to be both protective and toxic [75] in different systems.

In its physiological state, α-synuclein appears to be constitutively N-terminal acetylated [76] at several lysines residues. The presence of this PTM appears to affect the binding to high curvature vesicles and to vesicles owing low negative charge, whereas the binding to highly negative charged structures appears to be unaffected [77]. Overall, acetylation contributes to maintain the equilibrium between monomeric-, soluble forms and tetrameric-, membrane bound assemblies.

Another physiological PTM of α-synuclein is its C-terminal truncation [78, 79], associated with the lysosomal pathway. Carboxyl-terminal truncated α-synuclein has been estimated to constitute 10–25% of the LBs [79]. Indeed, C-terminal truncation has been associated with an increase in self-assembly properties of α-synuclein [78].

Nitration is another common PTM found in LBs [79]. Nitric oxide is produced upon oxidative stress, causing the nitration of tyrosine residues. Nitration of tyrosine residues in α-synuclein is supposed to hinder the formation of aggregates [80], causing the formation of dityrosine bridges that stabilizes low molecular weight oligomers.

When purified from LBs, α-synuclein appears to be mono-ubiquitinated [81]. The addition of a single ubiquitin molecule is a signal for proteasome degradation, whereas poly-ubiquitination triggers the degradation through the lysosomal pathway [82, 83]. However, α-synuclein may also be conjugated with the small ubiquitin like modifier (SUMO) in a process termed SUMOylation [84]. The addition of this residue was shown to have opposite effect to ubiquitination [85]. Whereas ubiquitination favours the degradation of α-synuclein, SUMOylation was shown to inhibit synuclein ubiquitination, leading to an increase of the local concentration of α-synuclein and therefore promoting the aggregation process.

Lastly, the presence of other proteins may affect the aggregation process. Although homotypic seeding is the preferred form of aggregation, heterotypic cross-seeding of α-synuclein with other natively unfolded proteins (such as tau or PrP^C^ [86, 87]) has been reported as well.

Physical factors that may impact amyloidogenesis are ascribed to thermodynamic events. Temperature, concentration and the application of an external force may increase the entropy of the system, hence increasing the chances of triggering the aggregation.

The sum of biological and physical factors dramatically impacts the experimental detection of the misfolding and aggregation process. Whereas the presence of aggregates has been classically studied by forcing the formation of fibrils in vitro [88, 89], the most recent techniques take advantage of the prion-like mechanism to seed the conversion, and thus reducing the time for the detection of the aggregates. Indeed, the presence of a single misfolded unit enhances dramatically the aggregation by the transmission (in vitro and in vivo) of its conformational information to natively folded neighbour molecules, giving rise to self-propagating molecules. The misfolding and prion-like conversion behaviour of one or more proteins has been observed in a wide variety of neurodegenerative diseases [8, 14, 15, 35]. Currently, the conversion behaviour of misfolded proteins (e.g. α-synuclein) is the basis for the development of a group of methodologies analyzing the process of protein misfolding in vitro.

### Protein misfolding cyclic amplification assays

**Fig. 1:**
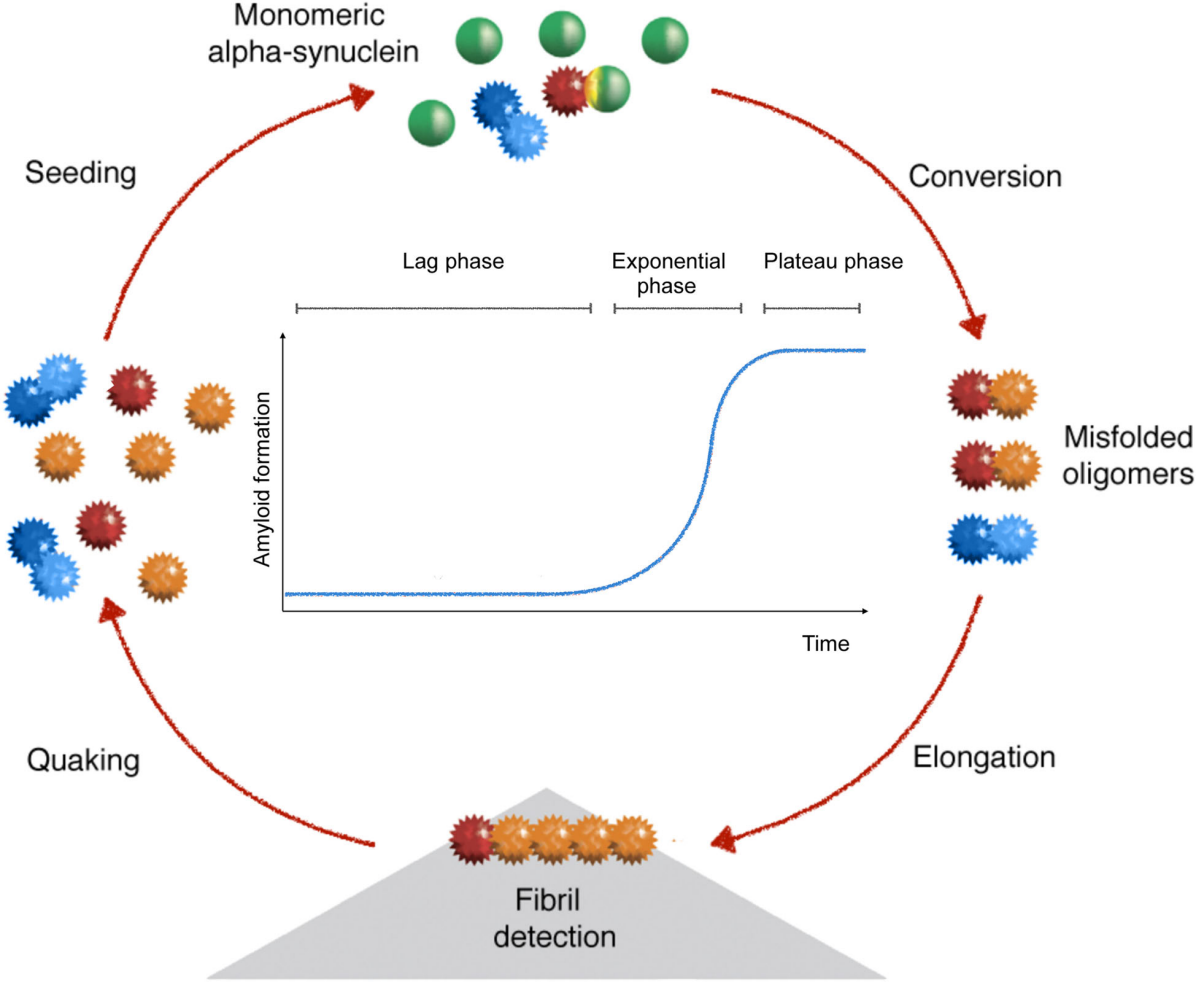
Diagram of the potential seeding-conversion mechanism of α-synuclein during the RT-QuIC. The seed (in red) triggers the aggregation of monomeric α-synuclein (substrate, in green). The conversion causes the conformational modification into misfolded oligomers (blue) that elongate into fibrils. After the detection of fibrils, a quaking event breaks the longer fibrils into shorter, reactive oligomers, which further seed the conversion of monomeric α-synuclein (modified from [92]). The classical shape of the kinetic curve is shown within the aggregation process

The importance of the self-assembly properties in the onset of neurodegenerative diseases have led to the investigation of the kinetic of the aggregation. During the onset of the pathology, this process is typically slow. However, it can be mimicked in vitro and, under appropriate conditions, it may occur much faster than during pathology. Independently from physical and chemical factors, which may influence the fibrillization process, the presence of a molecule which is prone to aggregation and can act as a nucleus or seed for the fibrillization, allows the reduction of the lag phase and avoids the possible denaturation of the monomers during the reaction [90, 91]. The growth of the α-synuclein fibrils follows a sigmoidal curve (Fig. 1). It displays a lag phase, during which the nucleation process subsequently, the exponential phase indicates the elongation of the fibrils, before a plateau phase can be observed once the monomers have been converted into β-sheet enriched species, which saturates the signal (Fig. 1). The seeded conversion assays have been applied for the detection of misfolded proteins for the diagnosis of prion diseases. In this frame, a fundamental innovation was the development of the protein misfolding cyclic amplification (PMCA) [93]. The aim of the study was to reproduce the conversion of PrP in vitro by seeding the reaction with minute amounts of the misfolded form of PrP. To this end, the aggregation of PrP^C^ from healthy brain homogenates was triggered by the addition of brain homogenates from scrapie-infected hamsters in a cyclic reaction, consisting in cycles of incubation and sonication. The former step allows the seed to contact and convert its native counterpart and to form growing oligomers, whilst the latter step breaks longer fibrils to increase the number of smaller seeding-competent aggregates, hastening the conversion process. The end-product was shown to be proteinase K (PK)-resistant by Western blotting. The PMCA was further automated [94–96] to make it suitable for the analyses of biological fluids (blood, urine or the cerebrospinal fluid (CSF) [97, 98]) and, recently, its application was expanded to the detection in biofluids of other neurodegenerative-related molecules, such as the CSF from patients with α-synucleinopathies [99].

To overcome the inherent complications of the PMCA, such as poor reproducibility, the time-consuming need of PK-resistant PrP^res^ detection via Western blotting, the careful handling of the infectious end-product and the lack of consistency with different prion strains [100], other in vitro*,* cell-free, amplification assays for misfolded proteins have been developed. The substitution of sonication by shaking [101, 102] represented a major advancement. Indeed, by increasing the air-water interface, shaking is thought to cause the unfolding of PrP and to enhance the fragmentation into oligomers, thus favouring the seed-substrate interaction [103]. The assay, termed real-time quaking-induced conversion (RT-QuIC), was further refined by the real-time screening of the course of the reaction by adding the fluorescent dye Th-T (Fig. 1). In this assay, small amounts of misfolded PrP^Sc^ seeds recruit single recombinant PrP^C^ substrate molecules and induce their conversion by integrating them into a growing amyloid aggregate concomitant with a conformational change of the substrate into a seeding-competent state (Fig. 1) [92, 101]. The RT-QuIC consists of cycles of incubation steps, where the size of the amyloid product, PrP^Res^, increases exponentially and vigorous shaking steps, which fragment PrP^Sc^ aggregates into smaller seeds promoting the conversion (Fig. 1). The PrP^Sc^-seeded conversion reaction product, PrP^Res^, is enriched in β-sheet secondary structures. The generation of these β-sheets can be monitored in real-time by using a temperature-controlled shaking fluorescence plate reader and by adding a fluorescence dye, Th-T, in the reaction mix.

### Development of the α-synuclein RT-QuIC

In contrast to previous diagnostic assays (colorimetric ELISA, Western blot etc.) for analysis of surrogate biomarker proteins (such as tau and 14–3-3), whose regulation in CSF is based on neurodegeneration process in brain tissue, RT-QuIC analysis allows an ultrasensitive (femtomolar) detection of pathogenic proteins, exhibiting a specificity close to 100% [92]. Recently, the RT-QuIC was successfully applied for the template-induced seeding conversion of α-synuclein, which enables the detection of α-synuclein misfolded species reported by different groups, employing different protocols in regards of the physical conditions (e.g., shaking, temperature, chaotropic agents) and chemical micro-environment (e.g., pH, ionic strength, presence of detergents).

*Fairfoul* and colleagues detected by RT-QuIC the seeding activity of misfolded α-synuclein derived from both CSF and brain homogenates [104]. The reaction was promoted by the addition of zirconia/silica beads to achieve sensitivity of 95% and specificity of 92–100%, respectively for DLB and PD diagnosis. Further, the authors reported the detection of prodromal stages of DLB, showing positive RT-QuIC results from cases affected by rapid eye movement (REM) behaviour sleep disorder, a hallmark of the prodromal stage of DLB. A biochemical and biophysical characterization of the RT-QuIC products from RT-QuIC reactions seeded with DLB derived brain homogenates was conducted by *Sano* and co-workers [105]. They obtained an accuracy of 100% specificity, 92% sensitivity for DLB and 95% sensitivity for PD. The tested α-synuclein was either wild-type, phosphorylated at residue 129 (pSer129) (commonly observed in patients with α-synucleinopathies) or non-phosphorylable α-synuclein, containing a point mutation (S129A). The seeding activity of S129A oligomers was higher than that of wild-type oligomers regardless of phosphorylation state. In a second RT-QuIC passage (that is, using the RT-QuIC products as seed for a second run), the highest seeding activity was observed when oligomeric species were produced by S129A α-synuclein in the presence of ATP and casein kinase (CK, that phosphorylates α-synuclein at residue S129), whereas wild-type α-synuclein in presence of either CK alone or both CK and ATP, showed a lower aggregation signal. These results point toward a possible protective effect of pSer129, which may generate inert fibrils faster hindering the aggregation. However, the presence of ATP alone was not investigated, as the presence of the nucleoside may promote the aggregation regardless the primary sequence of the substrate.

Another group, *Groveman* et al. [106], reported an aggregation of monomeric α-synuclein in reactions seeded with CSF or brain homogenate from DLB and PD patients. 100% of specificity and 93% of sensitivity for α-synucleinopathies (DLB and PD grouped together) was obtained by using as substrate α-synuclein owing a point mutation, K23Q, which confers a lower propensity to self-aggregation.

*Manne* and co-workers [107] achieved α-synuclein aggregation in the RT-QuIC by using as seed either CSF or brain homogenates in combination of SDS and chaotropic agents (zirconia/silica beads, similarly to *Fairfoul’s* protocol) to enhance the conversion of monomeric α-synuclein in RT-QuIC reactions, seeded with DLB and PD brain homogenates, with PD CSF and with a subset of progressive supranuclear palsy (PSP, a neurodegenerative disease characterized by tau aggregates). Moreover, researchers induced α-synuclein aggregation in reactions seeded with protein extracts from a microglial cell culture infected with pre-formed α-synuclein fibrils. The observations indicate that the seed for the RT-QuIC can also derive from a cell system.

*Candelise* and co-workers recently published a study in which positive RT-QuIC signals could be obtained specifically from DLB-brain seeded reactions [108]. In order to avoid possible contamination from macromolecules that may skew the aggregation process, a new pre-analytical fractionation protocol for brain homogenates was established. Under avoidance of bead addition (artificial inducer of α-synuclein aggregation), the α-synuclein RT-QuIC was successfully applied for the specific discrimination of DLB patients (positive response) from PD (negative response on control level). Biochemical analyzes confirmed the generation of a fibrillary, proteinase K (PK)-resistant seeding competent α-synuclein species only in DLB-brain seeded RT-QuIC reactions, pointing toward the existence of different α-synuclein strains in DLB and PD cases. Importantly, the RT-QuIC results were neither influenced by the brain region analyzed nor by the co-pathology or other epidemiological factors, such as age, sex, severity of the disease, and age of onset [108]. Controversial findings regarding a lack of α-synuclein seeding in PD patients might be explained by the addition of beads and the pre-analytical treatment (fractionation protocol) [104, 106]. Further factors, such as the kind of purification method of recombinant α-synuclein substrate or the presence of additional compounds (e.g. SDS, metals) may also have an impact on the seeding response.

The standard purification protocol includes a HPLC/FPLC step [105]. This step is particularly important for removing nucleic acids, as the presence of DNA/RNA influences the aggregation kinetics. Moreover, α-synuclein is prone to bind metal ions, specially copper (e.g. His50) which accelerate its aggregation. Therefore, standard protocols to purify recombinant α-synuclein from *E. coli* include chelator compounds, commonly EDTA, in the purification buffers. Finally, there are different purified α-synuclein species generated during the purification process (oligomeric and monomeric). Specifically the oligomeric species (removable by an additional filtration step) is prone to aggregation which results in a higher seeding propensity in the RT-QuIC as well as in self-aggregation of the substrate.

*Garrido* and co-workers [109] applied the protocol originally proposed by *Fairfoul* [104] and used in other studies [110, 111] to assess the presence of α-synuclein aggregation competent species in the CSF derived from α-synucleinopathies patients carrying a genetic PD mutation in the gene coding for leucine -rich repeat kinase 2 (LRRK2). By testing 10 idiopathic PD cases, they found a specificity of 90% and a specificity of 80%. Samples derived from LRRK-PD subjects showed a specificity of 40% (6 out of 15), while 3 of 16 (18.8%) of non-manifesting LRRK2 carriers showed a positive RT-QuIC signal response.

Finally, *De Luca* et al [112] performed a cross-sectional observational study, carried out by seeding the RT-QuIC reaction with brain material derived from cases affected by PSP, frontotemporal dementia (FTD) and corticobasal degeneration (CBD), PD and MSA. Serial steps of high speed centrifugation were conducted to enrich the seeding-competent α-synuclein species and diluted to a working concentration of 10^− 3^. Moreover, they obtained 47 samples of olfactory mucosa (OM) from PD, MSA, PSP and CBD cases. Researchers were able to detect seeding activity from reactions seeded with brain homogenates from PD and MSA patients, while no signal could be detected from reactions seeded with PSP, CBD, FTD or controls. When testing the OM samples, they detected seeding activity in 10 out of 18 PD cases (55.5% sensitivity) and in 9 out of 11 MSA cases (81.8% sensitivity). Thus, the overall sensitivity of PD and MSA grouped cases was 65.5%, whereas negative controls from PSP and CBD gave positive results respectively in 1 out of 6 and 2 out of 12 cases (overall specificity 84.3%). Both brain -and OM seeded reactions showed a lag phase of approximately 40 h. The group further analyzed the RT-QuIC fibrils by transmission electron microscopy, considering the length, the shape and the number of fibrils in the RT-QuIC end-products seeded with OM derived material. They found a statistically significant difference between PD derived fibrils and MSA derived fibrils, with the former owing a shorter over-twist distance compared to the latter. These fibrils were different from those derived from the positive RT-QuIC assays seeded with PSP and CBD material (respectively, PSP derived were longer than MSA, whilst CBD were the shortest produced during the RT-QuIC).

Thus, outside of the great improvement in the usage of a seed derived from a source other than CSF (which dramatically improves the well-being of a patient, compared to the painful procedure of the lumbar puncture), they also produced evidences of a difference in the seeding activity of PD derived α-synuclein compared to MSA derived α-synuclein, indicating a putative strain difference between both pathologies, in line with previous studies reporting putative strain differences among synucleinopathies [113–115].

Even though several studies (mentioned before) observed a seeding activity of pathogenic α-synuclein, their protocols were different for chemical and physical conditions and, more importantly, for the starting seed and the kind of purified α-synuclein substrate used for the reactions. Most of the groups used human full length α-synuclein from different companies, and different kinds of beads were added in the reaction to accelerate the conversion. In addition, the purified α-synuclein substrate can already be partially aggregated (preformed fibrils) or genetic (PD mutations were included in the substrate sequence), enhancing the seeding propensity in the RT-QuIC reaction. The presence of the positively charged histidine moiety (required for the purification), may further promote the self-aggregation properties of α-synuclein. Moreover, the addition of further compounds such as SDS can change the seeding kinetic and the sensitivity of the α-synuclein RT-QuIC. Differences among protocols and their clinical accuracy (specificity, sensitivity) are summarized in table form (Table 2).

**Table 2:**
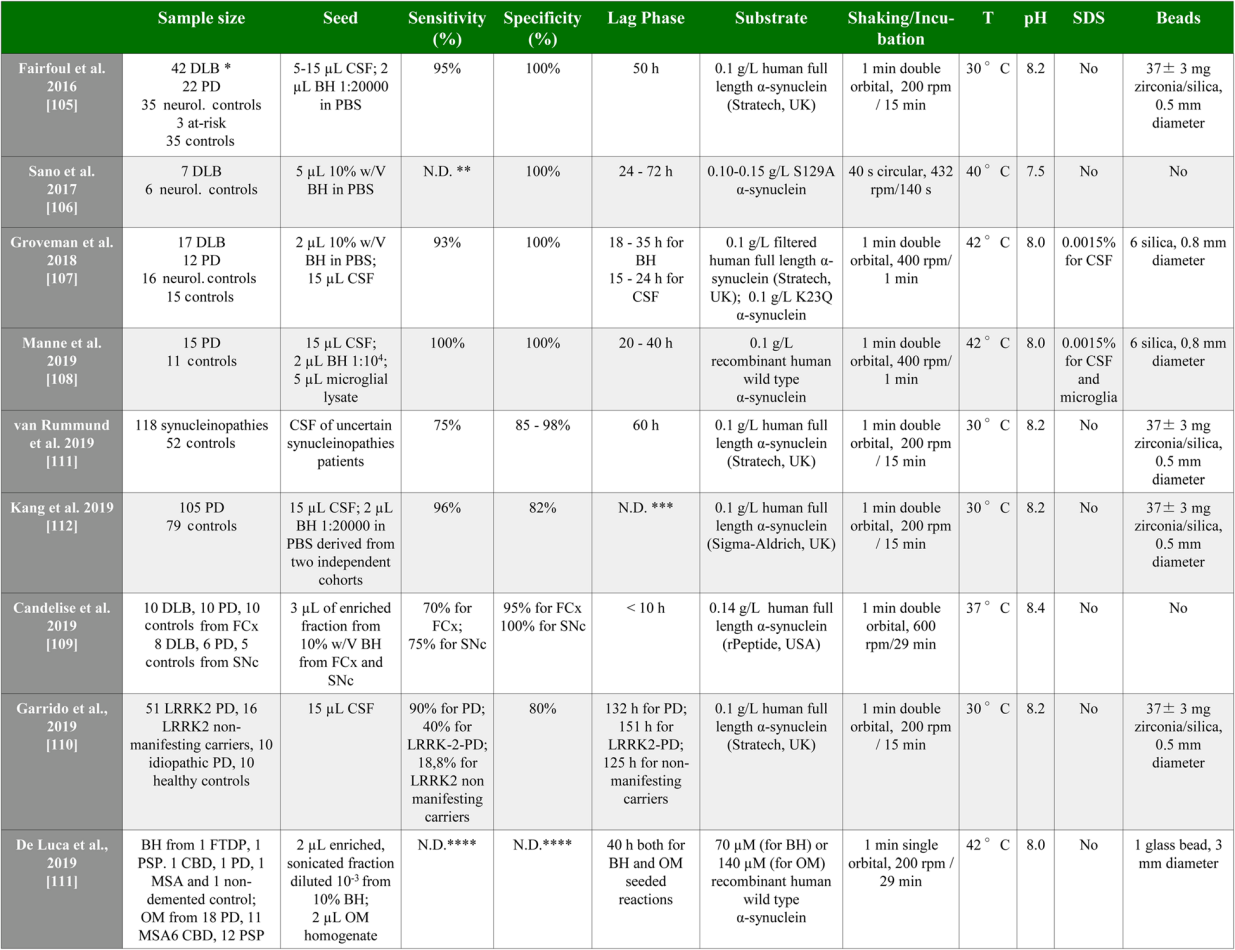
Comparison of different α-synuclein RT-QuIC protocols. The table summarizes the differences in the protocols so far developed to detect α-synuclein seeding activity in the RT-QuIC. In *Fairfoul* et al.*,* * 42 DLB samples included 12 pure DLB, 17 DLB + AD and 13 AD with incidental Lewy bodies. ** Not determined. *** In Kang et al., T_50_ (time to reach 50% of the maximum of fluorescence) was calculated as function of the unified Parkinson disease rating scale (UPDRS) instead of the lag time. T_50_ was approximately 50 h. **** Due to the limited number of samples analysed, *De Luca* et al. decided both to calculate specificity and sensitivity. Extracting these data from the paper resulted in specificity of 65.5% and sensitivity 84.3% for OM seeded reactions

It is therefore apparent that multiple experimental settings may be exploited for the detection of α-synuclein seeding activity in the RT-QuIC. A different “micro-environment” in the reactions, different physical conditions for the aggregation, a different source for the seed, the usage of different substrates or more probably the combinations of these factors, may lead to the detection of α-synuclein seeding activity from different α-synucleinopathies.

Upon protein aggregation, a population of conformational variants could be produced, which compete for the same source (i.e., the native version of the misfolded protein) as a function of the environmental conditions. The different conformations that arise from the same misfolded protein have been referred as strains.

### Evidences for the existence of different α-synuclein strains

The term “strain” was first introduced to discriminate the pathological phenotypes associated with the intracerebral inoculation of the prion protein in goats [116–118]. Upon misfolding, a single unit may accommodate into different conformations, giving rise to different pathological frames. Strains hence refer to separate conformations of the same protein. Since their inception in the prion field, a variety of strains has been characterized [119, 120]. The conformation may be altered by the genetic background of the host and the source of the pathogenic protein, as well as post-translational modifications, chemical and physical conditions of the micro-environment in which the process takes place, leading to a heterogeneous strain population.

#### Different transmission properties of α-synuclein species in vitro and in vivo

The transmissibility of misfolded α-synuclein (either from brain homogenates of a donor or recombinantly produced fibrils) indicates a prion-like seeding and conversion activity. Relevant for a successful transmission of α-synuclein fibrils in rodent brains inducing a Parkinson-like pathology is an overexpression of α-synuclein which serves as a substrate for the inoculated α-synuclein seed [121].

The transmissibility of MSA derived α-synuclein was demonstrated by the injection of brain homogenates from MSA patients into hemizygous TgM83^+/−^ mice [111], which possess the mutation A53T, associated with familial forms of PD [122].

TgM83^+/−^ rapidly developed signs of synucleinopathy and widespread deposition of LBs upon intracerebral inoculation of brain extracts from MSA cases. However, fibrils extracted from TgM83^+/+^ (homozygous for the mutation, naturally developing α-synuclein aggregates) were not able to passage the pathology when injected in TgM83^+/−^ mice. The same group reported in a follow up study [123] that fibrils derived from brain homogenates derived from PD patients were not able to trigger the pathology when injected into hemizygous mice or in HEK cells owing the same A53T mutation, similarly to what could be observed with fibrils derived from TgM83^+/+^ mice. These results indicated that different strains of α-synuclein underlie the clinical manifestations of PD and MSA.

Moreover, brain homogenates derived from DLB and MSA patients also differed in their seeding activity when injected into wild-type mice [113]. In addition, MSA derived α-synuclein is more aggressive than PD/DLB, which may depend on the conformation of the misfolded seed (more compact structure) as well as from the intracellular environment [115]. MSA- and PD/DLB α-synuclein have no cell type preference in seeding α-synuclein pathology, even though they showed a different cell type distribution in MSA and DLB. In transmission studies (carried out in TgM83+/− mice that were hemizygous for the human α-synuclein A53T transgene) with MSA α-synuclein there is a predominance of neuronal over oligodendroglial inclusions, which might be a consequence of the “artificial environment” not reflecting the conditions in MSA patients. In this context it was reported that the cellular milieu in oligodendrocytes is responsible for the transformation of misfolded α-synuclein into a MSA strain [115], while seeding activity of MSA-α-synuclein is maintained when propagated in neurons, which shows that the strain characteristics depend also on the conformation of the misfolded seed [115].

The first evidence of the ability of α-synuclein aggregates to produce different strains was performed by incubating monomeric α-synuclein in different salt concentrations [124].

High salt (termed buffer A) resulted in the formation of assemblies within hours, whereas low salt buffer (buffer B) had a longer lag phase before aggregation. Aggregate formation from buffer A was reversible (they depolymerize by incubation at 4 °C and re-polymerize upon heating at 37 °C). In contrast, aggregates formed in buffer B were irreversible.

Electron microscope analysis revealed differences in the structure of these aggregates. Assemblies from buffer A showed a cylindrical aspect, while aggregates derived from buffer B had a planar and often twisted structure. Due to their appearances, buffer A aggregates were termed fibrils and buffer B assemblies were termed ribbons. These two species were shown to further differ for their PK banding profile. Different PK profiles are generated due to different cleavage sites of the PK, which may depend on the conformation of misfolded α-synuclein. Indeed, ribbons could not bind to Th-T, whereas fibrils did. The structure of these aggregates also differs, with ribbons owing a rigid and β-sheet enriched N-terminal moiety, whereas the N-terminal part of the fibrils appears to be mostly disordered [124]. The two strains were further distinguished by their toxicity upon administration into human neuroblastoma SH-SY5Y cell cultures, with ribbons causing a less extensive cell deaths compared to fibrils. In a following study [114], fibrils and ribbons were reported to bear pathogenic potential when injected in the substantia nigra of rats, with fibrils exhibiting the highest toxic effect compared to oligomers or ribbons, indicating that strains can be further differentiated by their cytotoxic effects. Lastly, serial sonication steps were reported to produce a synthetic α-synuclein strain which displays less ability in seeding the aggregation of α-synuclein in neuronal cultures compared to PFFs. However, this strain had a greater ability of seeding tau aggregation in vitro [86] but only a minor effect on tau aggregation in transgenic mice. The presence of lipopolysaccharides was further reported to produce different conformations of α-synuclein fibrils [125].

Although α-synuclein oligomers are considered as the main reactive species for the onset of neurodegeneration [21], there are evidences of stabilized protofibrils that are unable to form mature fibrils. These species are termed off-pathway oligomers, as they result in stable oligomers lacking in seeding activity [126]. Various types of off-pathway oligomers have been generated in vitro by the addition of molecules that allow the formation of kinetically stable molecules unable to further aggregate into fibrils. Several molecules have been shown to favour the formation of off-pathway oligomers. By varying the salt-, buffer- and pH environmental conditions, *Pham* and co-workers [127] showed that different dopamine redox products display various effects on in vitro α-synuclein aggregation. Specifically, whereas under acidic conditions dopamine is stable and inhibits the oligomerization, higher pH values cause the formation of redox catabolites of dopamine, which promote the formation of soluble, SDS-resistant, off-pathway oligomers. However, the same redox product, 5,6-dihydroxyindole, induces the formation of on-pathway oligomers under acidic conditions generating fibrils that are Th-T insensitive.

Peroxidation-derived reactive aldehydes, such as 4-oxo-2-nonenal (ONE), have been shown to produce off-pathway oligomers as well [128]. However, these α-synuclein oligomers exhibit a beta-sheet structure and appear to be able to induce the formation of fibrils with a reduced rate of fibrillation.

Lastly, α-synuclein off-pathway oligomers may be generated by the presence of the polyphenol epigallocatechin gallate (EGCG [129]), a potent inhibitor of the in vitro aggregation of huntingtin and α-synuclein.

**Figure 2:**
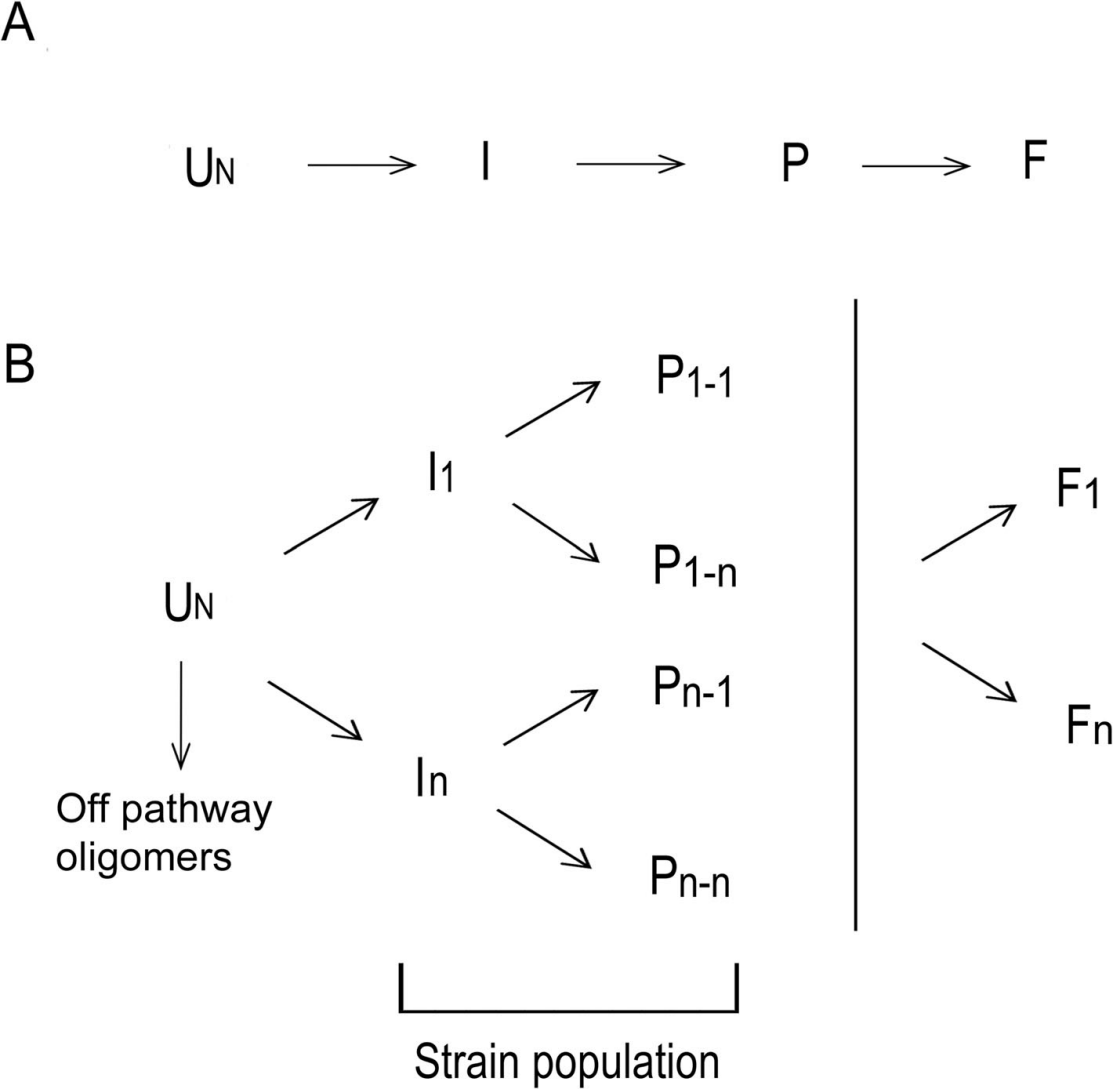
Proposed models for the aggregation pathway. **a** Established schematic view for the pathway of aggregation of natively unfolded proteins [40]. According to this model, from an unfolded protein (U_N_) is formed an intermediate (I), which further aggregates into protofibrils (P) and eventually into fibrils (F). **b** The extended model we propose takes into account the possibility that a population of strains are produced, together with off-pathway oligomers that do not show the ability to form α-synuclein fibrils. Intermediate forms marked as I_1_ to I_n_, each of them may produce a different protofibrillar form, marked as P_1-n_ to P_n-n_ (the protofibril P_1_ derived from the intermediate I_1_ until the protofibril P_n_ derived from the intermediate I_n_). It is noteworthy to state that the process may be in equilibrium between the aggregation of the intermediates and the rupture of protofibrillar forms. Further aggregation will cause the collapse into a limited number of fibrillar structures

The scheme for the aggregation pathway of natively unfolded proteins (Fig. 2A) may therefore be implemented (as shown in Fig. 2B) by taking into account the environmental factors and the production of a population of strains, whose constraints are dependent on the minimization of the free energy of the conformer and ultimately converge into a limited variety of fibrillary structures or off-pathway species (Fig. 2B) (in case of α-synuclein, fibrils, ribbons and amorphous aggregates [19, 40]).

## Conclusions

Since the introduction of the concept of self-propagating proteins as common feature in neurodegenerative diseases, whether different conformations, or strains of pathogenic proteinaceous material might underlie similar diseases has been a long lasting conundrum. As strains may emerge from light variations in the reaction micro-environment and from the binding to different partners, an in-depth characterization of both the reaction conditions and the presence of biomolecules appear to be necessary. Here we extend the classical view of protein aggregation to include strain variability and micro-environmental factors that may aid to understand the heterogeneity of conformational variants of a natively unfolded protein such as α-synuclein.

“Pure” α-synucleinopathies strikingly differ for their clinical manifestation, route of spreading and prodromic phase of the disease. As the technology of in vitro, cell-free systems, such as the RT-QuIC, progresses, subtle differences of the misfolded species of proteins that may contribute to neurodegenerative diseases are starting to manifest, indicating that strain-typing might be a relevant factor behind the clinical course of similar, albeit different diseases, as in the case of PD and DLB.

Here, we attempted to comprehensively review the complex landscape that regulates the folding and aggregation of α-synuclein, scouring through the intrinsic (i.e., mutations associated with pathology and PTMs) and extrinsic (micro-environmental) factors that influence the topology of α-synuclein both in pathology and physiology. These factors need to be taken into account in order to develop reliable assays for the diagnosis of α-synucleinopathies.

Indeed, to establish the α-synuclein RT-QuIC in the diagnostic of α-synucleinopathies (pre-mortem in CSF or post-mortem in brain) the following standardization steps will be required: 1) Pre-analytical sample treatments (type of storage tube), 2) harmonization of test-protocols (composition of the reaction mix, kind of α-synuclein substrate, incubation times and shaking etc.), 3) definition of a positive reaction, 4) determination of stability of the misfolded α-synuclein seed against storage conditions, and 5) investigation of the tolerance of the RT-QuIC against blood contamination.

## Data Availability

The datasets used and/or analysed during the current study are available from the corresponding author on reasonable request.

## Abbreviations

DLB: Dementia with lewy body
FC: Frontal cortex
GAG: Glycosaminoglycan chains
IDPs: Intrinsically disordered proteins
Lewy body dementia; LBs: Lewy bodies
MSA: Multiple system atrophy
PD: Parkinson’s disease
PFF: Pre-formed fibril
PK: Proteinase K
PrP^C^: Cellular prion protein
PrP^Sc^: Scrapie prion protein
PTMs: Posttranslationale modifications
REM: Rapid eye movement
rfu: Relative fluorescence units
RT-QuIC: Real-time quaking-induced conversion
SNc: Substantia nigra pars compacta
Th-T: Thioflavin-T
